# Effect of Bleaching and Thermocycling on Resin-Enamel Bond Strength

**DOI:** 10.1155/2015/921425

**Published:** 2015-12-29

**Authors:** Horieh Moosavi, Hamideh Sadat Mohammadipour, Marjaneh Ghavamnasiri, Sanaz Alizadeh

**Affiliations:** ^1^Dental Materials Research Center and Department of Operative Dentistry, Faculty of Dentistry, Mashhad University of Medical Sciences, Mashhad 91735, Iran; ^2^Dental Research Center, Department of Operative Dentistry, Mashhad Dental School, Mashhad University of Medical Sciences, Mashhad 91735, Iran

## Abstract

The aim of this study was to evaluate the effect of bleaching and thermocycling on microshear bond strength of bonded resin composites to enamel. Enamel slices were prepared from ninety-six intact human premolars and resin composite cylinders were bonded by using Adper Single Bond 2 + Filtek Z350 or Filtek silorane adhesive and resin composite. Each essential group was randomly subdivided to two subgroups: control and bleaching. In bleaching group, 35% hydrogen peroxide was applied on samples. Thermocycling procedure was conducted between 5°C and 55°C, for 3.000 cycles on the half of each subgroup specimen. Then microshear bond strength was tested. Methacrylate-based resin composite had higher bond strength than silorane-based one. The meyhacrylate-based group without bleaching along with thermocycling showed the most bond strength, while bleaching with 35% carbamide peroxide on silorane-based group without thermocycling showed the least microshear bond strength. Bleaching caused a significant degradation on shear bond strength of silorane-based resin composites that bonded using self-etch adhesive resin systems.

## 1. Introduction

Bleaching popularity and introducing the new bleaching products every year lead to many studies about the effects of these products on teeth and dental restorative materials. Effects of free radicals releasing during bleaching process on physicomechanical properties of restorations such as their colour, surface roughness, strength, hardness, and ion leakage are subjects of most scientists' discussions [1]. Conventional composites have marked polymerization shrinkage because of Bis-GMA monomers, but silorane-based composites showed less shrinkage, thus microleakage and its side effects are less. However a previous study showed that restorations of both materials were clinically acceptable after 5 years [2]. It was shown that bleaching of fluorosed teeth reduces bracket bond strength to enamel, but the bond strength with these still exceeds the minimum [3]. It has been stated that bond strength of restorations to enamel and dentin was affected by carbamide peroxide and this defect was related by carbamide peroxide concentration [4]. Just some articles showed bleaching agents could penetrate pulp chamber through composite restorations [5, 6]. In addition, it was suggested that bleaching could adversely affect the interfacial fracture toughness of dentin-resin composite adhesive interfaces [7]. The other studies showed increasing marginal leakage in postoperative bleaching of class V resin composite restorations [8, 9]. However, it was found that immediate postoperative bleaching with 20% carbamide peroxide gel, 6% hydrogen peroxide, and 19% percarbonate gel for 14 days had no influence on microleakage of Filtek composite bonded with Scotchbond 1 at the occlusal margins of the Class 1 restorations [10]. Composite materials may be failed by mechanical and thermocycling (TC) condition, interfacial debonding, microcracking, and filler particle fracture, which could reduce the survival probability of composite restorations in vivo [11]. Numerous in vitro studies of the mechanical performance of dental composite materials after TC showed that artificial aging protocols accelerate degradation of materials, which significantly degrades mechanical properties [11–16]. In contrast, Smisson et al. found no significant differences in the mechanical properties (flexural strength and bond strength) of a hybrid resin composite tested under five different TC protocols [17]. A recent review concluded that there was no standardized TC protocol to reproduce aging conditions in the oral cavity [12]. As far as the authors are aware, effects of contemporary bleaching and thermocycling on bonded composites were a few in previous studies, to date. Therefore, the aim of this study is to evaluate the effect of bleaching and thermocycling on microshear bond strength of bonded dimethacrylate and silorane-based resin composites to enamel. The null hypotheses were that the bonded composites are not significantly influenced by (1) type of resin composite or (2) bleaching procedure or (3) thermocycling regimen.

## 2. Materials and Methods

### 2.1. Specimen Preparation

**Figure 1:**
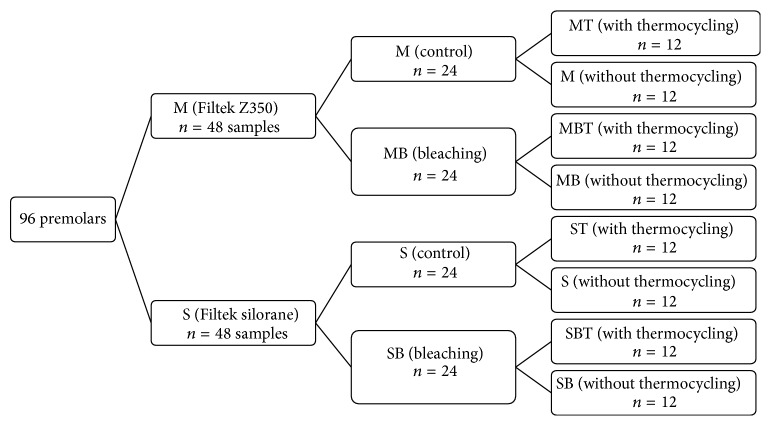
The schematic of experimental groups.

Ninety-six intact premolars had been extracted for orthodontic reasons that were gathered following informed consent approved by the Commission for Medical Ethics of the University of Medical Sciences (N#901114). The remaining soft tissue, calculus, and plaque were removed with rubber cap and slurry of pumice after hand scaling instrument and then stored in 0.1% thymol solution until operation time. One millimeter thickness of enamel slices was prepared parallel to proximal surfaces and polished by 200, 400, and 600 grit silicon carbide papers (3M, St. Paul, USA) under water cooling, respectively. Samples were divided into two groups based on resin composite types (*n* = 48); nanofill resin composite with dimethacrylate base (3M ESPE, Filtek Z350 Universal Restorative Dental Products, St. Paul, USA) (group M) and silorane-based resin composite (3M ESPE, Filtek silorane, Dental Products, St. Paul, USA) (group S). For the groups restored with the dimethacrylate material (group M), enamel surface was etched by 37% phosphoric acid gel for 30 seconds (3M ESPE, Scotchbond Etchant, Dental Products, St. Paul, USA), rinsed with water/air syringe for 30 seconds, and dried gently with air syringe only for 5 seconds. Then, two consecutive coats of Single Bond adhesive (3M ESPE, Adper, Dental Products, St. Paul, USA) were applied on etched surface. Each layer was gently air dried for 5 seconds to make a delicate and shiny surface and light cured for 20 seconds by light cure device with 500 mW/cm^2^ intensity (Blue phase C8, Vivadent Ivoclare, Austria). The groups were restored with the silorane-based composite resin (Filtek silorane; group S), each Silorane System Adhesive Self-Etch Primer and Bond (3M ESPE, Dental Products, St. Paul, USA) was applied according the manufacture's instruction (Table 1). In all samples, before adhesive curing, silicon tubes with an internal diameter of 0.7 mm and a height of ~2 mm were placed on the adhesive surface and then the adhesives were light-cured. Composites were incrementally condensed in tubes, by a plastic instrument in two 1 mm layers and each layer was polymerized for 40 seconds. After 24 hours, silicon tubes were removed by using a scalpel (Fine science tools GmbH, Germany) with a number 11 blade (Fine science tools GmbH, Germany). Half samples of each resin composite were bleached by 35% hydrogen peroxide (Opalescence Endo, Ultradent Products, USA) which was used for 3*∗*20 minutes, according to manufacturer's instruction. So both resin composite types were divided into two subgroups (*n* = 24): control (C) and bleaching (B). The half of each resin composite subgroups (*n* = 12) was subjected to thermocycling (T) process (custom procedure made by NIOM-Scandinavian Institute for Dental Materials, Haslum, Norway) for 3.000 cycles between 5°C and 55°C in deionized water with a dwell time of 30 seconds and transfer time of 10 seconds (Figure 1). Samples were stored in artificial saliva in 37°C prior to microshear bond strength test. The shear-bond strength was measured with Universal testing machine (SANTAM STM-20, Santam, Iran). A knife edge shearing rod with a crosshead speed of 0.5 mm/min and 6 kg load cell was used. It was recorded the load at failure for each specimen. The microshear bond strength of the specimens were calculated and expressed in MPa. The failure mode of each beam was examined under a stereomicroscope (Dino-Lite Pro, AnMo Electronics Corp, Taiwan) at a magnification of up to 50x. Failures were categorized as cohesive (occurring within dentin or composite), adhesive (occurring between the two materials), or mixed (a combination of cohesive and adhesive).

### 2.2. Statistical Analysis

All statistics were performed with SPSS version 11.0.0 (SPSS Inc., Chicago, IL, USA). The Kolmogorov-Smirnov test confirmed the normal distribution of the data, and a parametric approach was used to verify the null hypotheses. Interaction among study variables (type of composite with regarding to adhesive, bleaching, and thermal cycling) was tested by ANOVA with Tukey's post hoc multiple-comparison tests. The significance level was set at *α* = 0.05.

## 3. Results

The mean and standard deviations of each group are displayed in Table 2. Maximum and minimum of bond strength were observed in groups MT and SB, respectively. According to analysis of variance there was an interaction among three variables: composite type, bleaching, and thermocycling. Thermocycling caused an increasing in shear bond strength of control groups and bleaching by 35% hydrogen peroxide groups. Bleaching for both resin composites led to lesser bond strength compared to without bleaching application.

Tukey's test results for subgroups M and S showed significant difference in shear bond strength of control and bleaching subgroups with or without thermocycling (*P* < 0.05) (Table 2).  Table 3 demonstrates that the highest number of cohesive failure in resin occurred in case of Filtek Z350, whereas the higher mixed failure was observed with Filtek silorane resin composite.

## 4. Discussion

Microshear bond strength has been characterized as the maximum stress that a material subjected to a shearing load can resist before failure. It concerns one measurement of bond strength for dental materials, as considerable shear stresses occur during the complex mastication procedure [18]. Using microshear test for evaluating the adhesive-tooth interface has some benefits, like lower requirements in specimen collection, facility in the preparation, and standardization of the test area by microtubes fabrication [19–21]. The findings of this in vitro study suggest that the evaluated factors (type of composite with regarding to adhesive, bleaching, and thermal aging) have a decisive influence on the microshear bond strength of bonded resin composites. Thus, null hypotheses are rejected. Variations in the mechanical properties of dental composites are likely due to differences in the chemical composition of the matrix, fillers, and filler size and distribution [22]. Therefore, in the current study we examined composite materials differing in matrix and filler composition, including a nanofilled composite, methacrylate-based (Z350), and microfilled hybrid composite, silorane-based (P90). Although filler volume of resin composites may have important role in mechanical properties, but the complex chemical composition of composites (matrix, filler size, distribution, and filler-resin coupling) is responsible for the different performance characteristics of these materials [23]. Degradation of composite resins in the oral environment is attributed to the resin matrix, filler particles, and hydrolytic instability of the silane coupling agent at the polymer-silica interface [24]. Effects of bleaching agents on physical properties of adhesive restorations remain controversial. Reports of the literature suggested that bleaching agents can remove SiO_2_ from inorganic components of sealing materials [25]. Slight significant increasing in surface roughness and porosity of microfilled and hybrid resin composites was reported after application of 10–16% carbamide peroxide [26]. These findings were not corroborated in previous study which was applying 6% hydrogen peroxide gel on a hybrid composite in a cycling protocol with intermittent storage of the samples in pooled human saliva [27]. It seems that saliva forms a protection layer on restorative materials which might have modified or attenuated the hydrogen peroxide impact. Albeit numerous studies discovered potential changes in the physical properties of resin composite restorations after bleaching, they could not show the clinical significance of these modifications and prescribed further clinical exploration [28]. The results of present in vitro study showed that the microshear bond strength of bonded composites significantly depends on the base type of composite and bleaching affected the composite-enamel shear bond strength. Additionally, methacrylate-based resin composites presented more bond strength than silorane-based restorations in each situation. This can be explained by the fact that the degree of subsurface polymerization, degree of conversion, and depth of curing in the methacrylate-based composite are more than silorane-based resin composites [20, 29, 30]. Also, self-etching adhesives exhibit lower bond strengths to enamel than do the etch-and-rinse systems, as described by some studies [31, 32]. In this manner, the weaker attaching to enamel may have created the lower shear bond strength values compared with the dimethacrylate framework in light of the fact that the bonded surface consisted of enamel. In addition, the silorane restorative system presents a different two-bottle adhesive system. The primer agent of this system is light-cured and, after the light activation, the bond agent is applied over the primer layer. Thus, the primer agent of this system is responsible for creating the hybrid layer, in contrast with the conventional self-etching adhesive systems, which form the hybrid layer with a mix of primer and bond agent. Accordingly, the adhesive of the silorane system delivers a tooth-resin composite interface that is made out of the tooth structure; a hybrid layer formed by the primer agent; a bond applied over the primer, functioning as an intermediate resin with low viscosity; and the composite resin. Because of this confused procedure, a feeble bonding between the two substrates referred to can trade off the bond strength of the silorane-based system. In this study, thermocycling procedure increased shear bond strength. This outcome was in contrary with previous studies which observed no changes in enamel bond strength of methacrylate-based composites after thermocycling and which stated aging did not affect silorane-based composite bond strength to dentin surface [33, 34]. It is accepted that the aging process and thermal shocks cause weakness of bonding surfaces or debonding of it because of different thermal expansion coefficient between dentin and composites which makes fatigue cycles in interface. Thermocycling process is accompanied by water absorption that makes changes in mechanical and physical properties of resin composites such as strength and hardness [35]. Also, water absorption and PH changes accredited breakdown of resin and silane bond between filler particles and resin. In this way, aged composites are more sensitive to load. Despite of all these explanations, our result can be clarified by this sentence: the heat of thermocycling procedure up to 3.000 cycles can enhance the degree of composite conversion and cause increasing of adhesion and bond strength. However, an important limitation of the present study is that only one thermocycling protocol was used, which complicates the analysis and comparison of responses of materials to physiological aging that may contain other stresses such as load and PH cycling. With respect to failure mode, cohesive failures were predominant for all tested groups of Filtek Z350 resin composite. The most predominant fracture pattern for silorane composite was mixed failure. This finding is in correspondence with the bond strength results. It seems that lower depth of cure, degree of conversion, and reduced polymerization below the surface for silorane-based composites compared to methacrylate-based composites may be responsible for high incidence of mixed failure in the resin composite. Further research is suggested to investigate other bleaching agents and methods on properties of aged or nonaged restorations accompanying load and PH cycling just as mouth simulation.

## 5. Conclusion

According to the results of this study and regarding limitations, methacrylate-based resin composite that is used with total-etch mode had higher microshear bond strength than silorane-based which uses a self-etch adhesive system under bleaching and thermocycling conditions. Bleaching affected a significant degradation on shear bond strength of silorane-based resin composites.

## Figures and Tables

**Table 1:** Materials, chemical compositions, and manufacturer used in the study.

Material	Components	Manufacturer
Filtek Z350 resin composite	BIS-GMA, BIS-EMA (6), UDMA, and TEGDMA, a nonagglomerated/nonaggregated, 20 nm nanosilica filler, loosely bound agglomerated zirconia/silica nan cluster, consisting of agglomerates of primary zirconia/silica particles with size of 5–20 nm fillers (82% wt)	3M ESPE, Filtek Z350 Universal Restorative Dental Products, St. Paul, USA

Filtek silorane resin composite (P90)	Silane-treated quartz, 3,4-epoxycyclohexylcyclopolymethylsiloxane, yttrium trifluoride, bis-3,4-epoxycyclohexylethyl-phenylmethylsilane, mixture of epoxy functional di- and oligo-siloxane by-products, mixture of alpha substituted by-products, mixture of other byproducts, and mixture of epoxy-mono-silanole byproducts (79% wt)	3M ESPE, Filtek silorane, Dental Products, St. Paul, USA

Scotchbond Etchant	32% phosphoric acid, fumed silica, and water	3M ESPE, Scotchbond Etchant, Dental Products, St. Paul, USA

Adper Single Bond 2	BisGMA, HEMA, dimethacrylates, ethanol, water, a novel photoinitiator system and a methacrylate functional copolymer of polyacrylic and polyitaconic acids, and silica particles	3M ESPE, Adper Single Bond 2 adhesive, Dental Products, St. Paul, USA

Silorane System Adhesive Self-Etch Primer	2-Hydroxyethyl methacrylate (HEMA), bisphenol A diglycidyl ether dimethacrylate (BisGMA), water, ethanol, phosphoric acid-methacryloxy-hexyl esters, silane-treated silica, 1,6-hexanediol dimethacrylate, copolymer of acrylic and itaconic acid, (dimethylamino)ethyl methacrylate, DL-camphorquinone, phosphine oxide	3M ESPE, Dental Products, St. Paul, USA

Silorane System Adhesive Bond	Substituted dimethacrylate, silane-treated silica, triethylene glycol dimethacrylate (tegdma), phosphoric acid methacryloxy-hexyl esters, 1,6-hexanediol dimethacrylate, and DL-camphorquinone	3M ESPE, Dental Products, St. Paul, USA

Opalescence Endo	35% hydrogen peroxide	Ultradent Products Inc., USA

HEMA: 2-hydroxyethyl methacrylate; Bis-GMA: bisphenol A diglycidyl methacrylate; TEGDMA: triethylene glycol dimethacrylate; BIS-EMA: ethoxylated bisphenol A glycol dimethacrylate; UDMA: urethane dimethacrylate.

The “number (6) and (90)” are as the chemical formulation or the part of name of “BIS-EMA” and “Filtek Silorane resin composite” respectively.

**Table 2:** Mean values and respective standard deviations of microshear bond strength (MPa) in experimental groups.

Experimental groups abbreviation	Mean (MPa)	Std. deviation	*N*	*P* value
Filtek Z350 resin composite (group M)				
MT	44.64a	11.62	12	<0.001
M	18.18b	5.68		
MBT	39.93a	11.03	13	
MB	21.09b	4.79		
Filtek silorane resin composite (group S)				
ST	26.33b	8.61	11	<0.001
S	16.56bc	3.55		
SBT	24.20b	7.60	12	
SB	9.73d	2.38		

*P* value	*P* < 0.05			

Significant changes in column for experimental groups are demonstrated by different letters for tests (Tukey's test *P* < 0.05).

**Table 3:** The fracture pattern mode % (*N*) of each experimental group.

Resin composite	Experimental groups description	Adhesive	Cohesive in resin	Cohesive in dentin	Mixed
Filtek Z350 resin composite (group M)	MT	16.7 (2)	58.3 (7)	8.30 (1)	16.7 (2)
	M	25.0 (3)	58.4 (7)	8.30 (1)	8.30 (1)
	MBT	16.7 (2)	75.0 (9)	0.00 (0)	8.30 (1)
	MB	16.7 (2)	50.0 (6)	16.7 (2)	16.7 (2)

Filtek silorane resin composite (group S)	ST	16.7 (2)	8.30 (1)	8.30 (1)	66.7 (8)
	S	8.3 (1)	25.0 (3)	16.7 (2)	50.0 (6)
	SBT	33.3 (4)	16.7 (2)	0.00 (0)	50.0 (6)
	SB	25.0 (3)	25.0 (3)	8.30 (1)	41.6 (5)
